# On a New Mode of Performing Iridectomy

**Published:** 1864-05

**Authors:** Julius Homberger


### ON A NEW MODE OF PERFORMING IRIDECTOMY.

By JULIUS HOMBERGER, M.D.

A great difficulty in performing Iridectomy, for the purpose of diminishing intra-ocular pressure, consists in the *removal of the iris to its ciliary insertion.* Another necessity, which is also not easily accomplished in many cases, is the *excision of a large piece* of the iris. If it is necessary to go far beyond the margin of the pupil with the lanceolar knife, in order to get a large corneal wound, the danger arises of injuring the lens, which is considerably pressed forward in glaucoma. Again, the cases are not rare where even experienced assistants do not cut off the iris to the edge, and thus cause a failure of the operation, or make at least its result very doubtful.

It is not my intention to criticize the different modifications which have been invented by Von Graefe, Arlt, Frœbelius, Bowman, and others, with a view to do away with these difficulties. No practical surgeon will deny, that, in spite of all modern propositions, the execution of Iridectomy is still attended by the above-named inconveniences. Therefore, though the method which I am going to describe has not yet stood the test of numerous experiments on living subjects, I do not hesitate to recommend it to the readers of this journal for trial, confiding in the easiness of its execution, and the certain results which it seems to promise.

With a cataract knife, the point of which, directed towards the centre of the globe, is pushed into the sclerotic at a distance of one line from the margin of the cornea, a linear opening is made, which, by merely pushing forward the knife, is lengthened in a radial direction, until the cut reaches threequarters of a line beyond the edge of the cornea. During the performance of this cut, the back of the knife does not for one moment leave its direction towards the centre of the eyeball. The knife is then gradually withdrawn, so that the aqueous humor is slowly evacuated. By this first act of the operation, the anterior chamber is opened, and the iris fissured from its ciliary insertion up to a point about half a line distant from its periphery.

The second act of the operation consists in the introduction into the wound of one branch of a fine, but strong, pair of scissors, slightly laterally curved. The point is introduced along the posterior surface of the cornea into the anterior chamber, and the cutting edge of the branch laid into the junction of iris and cornea. With one or two movements of the scissors, a wound is produced corresponding with the size of the piece of the iris which is intended to be removed. It will be necessary, in order to be able to introduce the scissors, to enter first but a little way into the wound made by the knife, and almost rectangularly to the latter, to make way for the branch of the scissors.

In the third act, a common iris-forceps is introduced into the anterior chamber, but not in a diagonal direction, as usually. With the point of the forceps the operator takes hold of that part of the iris next to the angle of the wound, and by a slight traction, in the direction of a tangent, touching the margin of the cornea in the wound, he tears the already fissured iris up to the pupilar margin, and then by continued pulling he severs it from its ciliary insertion. As soon as the iris is thus torn off up to the opposite angle of the corneal wound, the operator himself, or an assistant, removes the separate segment of the iris, with either knife or scissors.

The advantages of this method I wish to condense in the following points, and shall be happy if, by my proposition of a more convenient way of performing Iridectomy, I add a mite to the universal diffusion of this important operation.

1. The opening of the anterior chamber is made in such a way, that the instruments do not in any way come in contact with the pupillary region, and there is, therefore, no danger of wounding the lens.

2. The inner edge of the corneal wound is made with much more certainty in the junction of iris and cornea, than with either knife or lance.

3. The tearing of the iris from its insertion loses, by the previously made fissure in the iris, the danger of dialysis; while it insures, on the other hand, a peripherical pupil with more certainty than if the iris is cut off after having been dragged out in the manner hitherto practised.

4. The cutting off of the iris may be performed even by assistants of little experience; because, if ill-executed, it does not, as in the usual methods, render it frequently impossible to resume hold of the iris.

Finally, I may be permitted to remark, that I do not consider the division of some fibres of the ciliary muscle (Hancock) of great therapeutical importance; but that I think the angular opening, which allows a part, at least, of the aqueous humor to escape for some time, is very favorable to a gradual diminution of intra-ocular pressure. The importance of a compressive bandage during the after treatment may, by this circumstance, be considerably lessened, or even totally annulled.

